# Developing an in vivo porcine model of duct‐to‐mucosa pancreaticojejunostomy (Yonsei‐PJ^DTM^)

**DOI:** 10.1002/ags3.12310

**Published:** 2020-01-28

**Authors:** Munseok Choi, Chang Moo Kang

**Affiliations:** ^1^ Division of Hepatobiliary and Pancreatic Surgery Yonsei University College of Medicine Seoul Korea; ^2^ Department of Surgery Yonsei University College of Medicine Seoul Korea; ^3^ Pancreaticobiliary Cancer Clinic Yonsei Cancer Center Severance Hospital Seoul Korea

**Keywords:** education, laparoscopy, pancreaticoduodenectomy, pancreaticojejunostomy

## Abstract

Laparoscopic pancreaticoduodenectomy (LPD) is technically feasible, but its safety is still controversial. Pancreas texture and the small size of the main pancreatic duct indicate laparoscopic pancreaticoduodenectomy (LPD) as a challenging procedure. Thus, LPD could be a risk factor for postoperative pancreatic fistula (POPF), longer hospital stay, and delayed adjuvant chemotherapy that affects long‐term oncologic outcome. So, it is important to promote education on LPD especially techniques for pancreaticojejunostomy. A porcine model for duct‐to‐mucosa pancreaticojejunostomy (PJ) (Yonsei‐PJ^DTM^) was developed, and details of the model will be described in this report.

## Graphical abstract

In vivo porcine model of duct‐to‐mucosa pancreaticojejunostomy developed and details of the porcine model will be described in this report.

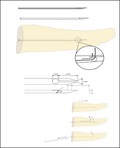

## 1 INTRODUCTION

Technical feasibility and surgical experiences of laparoscopic pancreaticoduodenectomy (LPD) have been reported, but the safety of LPD is still controversial. In fact, a recent randomized control study comparing LPD and open pancreaticoduodenectomy (OPD) raised considerable safety issues about LPD.^1^ Pancreaticoduodenectomy (PD) has two phases; *resection* and *reconstruction*.^2^ LPD is not that easy of a procedure. For example, in periampullary cancers associated with cholangitis and pancreatitis, resection will be difficult due to severe adhesion and invasion associated with potential risk of combined vascular resection. On the other hand, reconstruction is expected to be easy due to enlarged bile duct and remnant hard pancreas with dilated pancreatic duct. However, these clinical circumstances will be the opposite in benign and low‐grade malignant periampullary neoplasm.^3^

In particular, managing the remnant soft pancreas with small pancreatic ducts is believed to be one of the challenging procedures during LPD. This is because it is regarded as a risk factor to clinically relevant postoperative pancreatic fistula (POPF),^4^ resulting in prolonged hospital stay, increased medical costs, and even surgery‐related mortality. These factors also apply to OPD. Therefore, surgical simulation is very important to successfully manage the remnant soft pancreas with small pancreatic duct during PD, especially for less experienced pancreatic surgeons.^5^ It is agreed that that soft remnant pancreas with small pancreatic duct is risky, and safe pancreaticojejunostomy (PJ) is vital during PD. However, there are no appropriate ways to develop surgical skills for this challenging procedure. Surgeons hope to overcome the learning curve and become fluent in surgical techniques for safe PJ based on accumulated surgical experience. This is a *critical unmet need* in the clinical practice of pancreatic surgery.

In this report, a newly developed porcine model for duct‐to‐mucosa PJ (Yonsei‐PJ^DTM^) is described. This porcine model is quite similar to that for human soft remnant pancreas with a small pancreatic duct.^6^ We believe that every new surgeon who needs to perform PD or prepare for PD, regardless of whether they are using the minimally invasive or open approach, can benefit from the present model. The method teaches not only the concepts of the surgical procedure, but also improves surgical skills for safe PJ.

## 2 MATERIAL AND METHODS

### 2.1 Preparation of porcine model for PJ (Yonsei‐PJ^DTM^)

#### 2.1.1 Model concepts

The following is background for developing a porcine PJ model. Implantation of an artificial neo‐pancreatic duct is the main idea of the Yonsei‐PJ^DTM^ model.

1. In the porcine model, dissection of the pancreatic neck above the superior mesenteric vein‐splenic vein‐portal vein confluence is very similar to that in real patients.
2. When dividing the pancreatic neck, the left‐sided porcine pancreas is soft and appropriate for simulating remnant soft pancreas with a small pancreatic duct in PD.
3. However, the actual pancreatic duct of the porcine pancreas is too small to be appropriate for duct‐to‐mucosa (DTM) anastomosis training.^7^
4. Therefore, an artificial tube with small lumen can be implanted into the left‐side porcine pancreas after division of the neck for simulating soft remnant pancreas with a small pancreatic duct.

### 2.2 Preparation of the Yonsei‐PJ^DTM^ model

**Figure 1:**
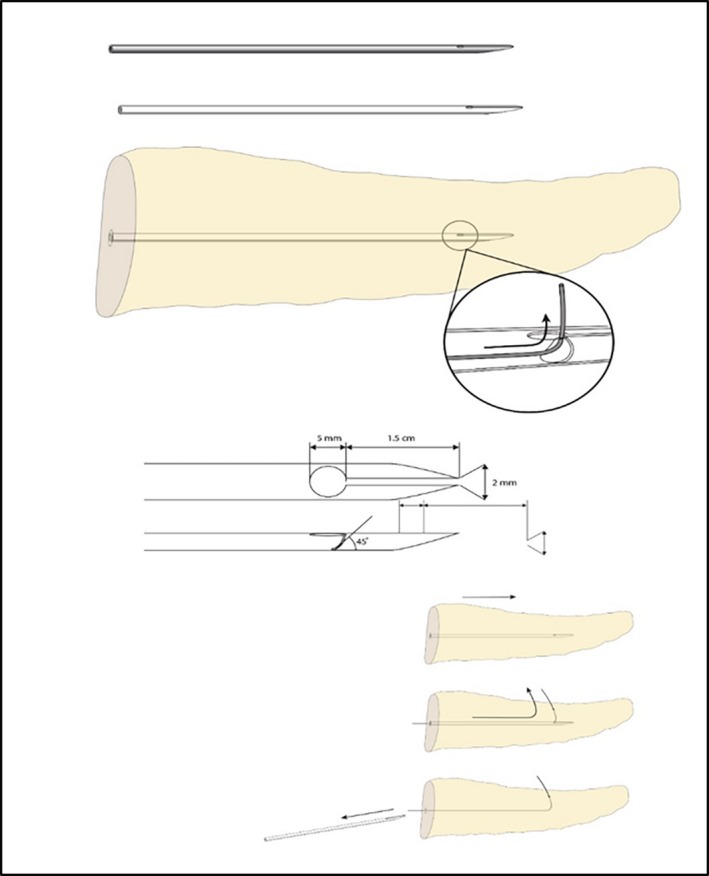
Specially designed catheter for preparation of a Yonsei‐PJ^DTM^ model. Using this specially designed catheter, the direction of the guidewire can be changed to penetrate the dorsal pancreas. The catheter can be removed with a left‐side penetrated and positioned guide‐wire, through which a subsequent artifical neo‐pancreatic duct can be inserted

**Figure 2:**
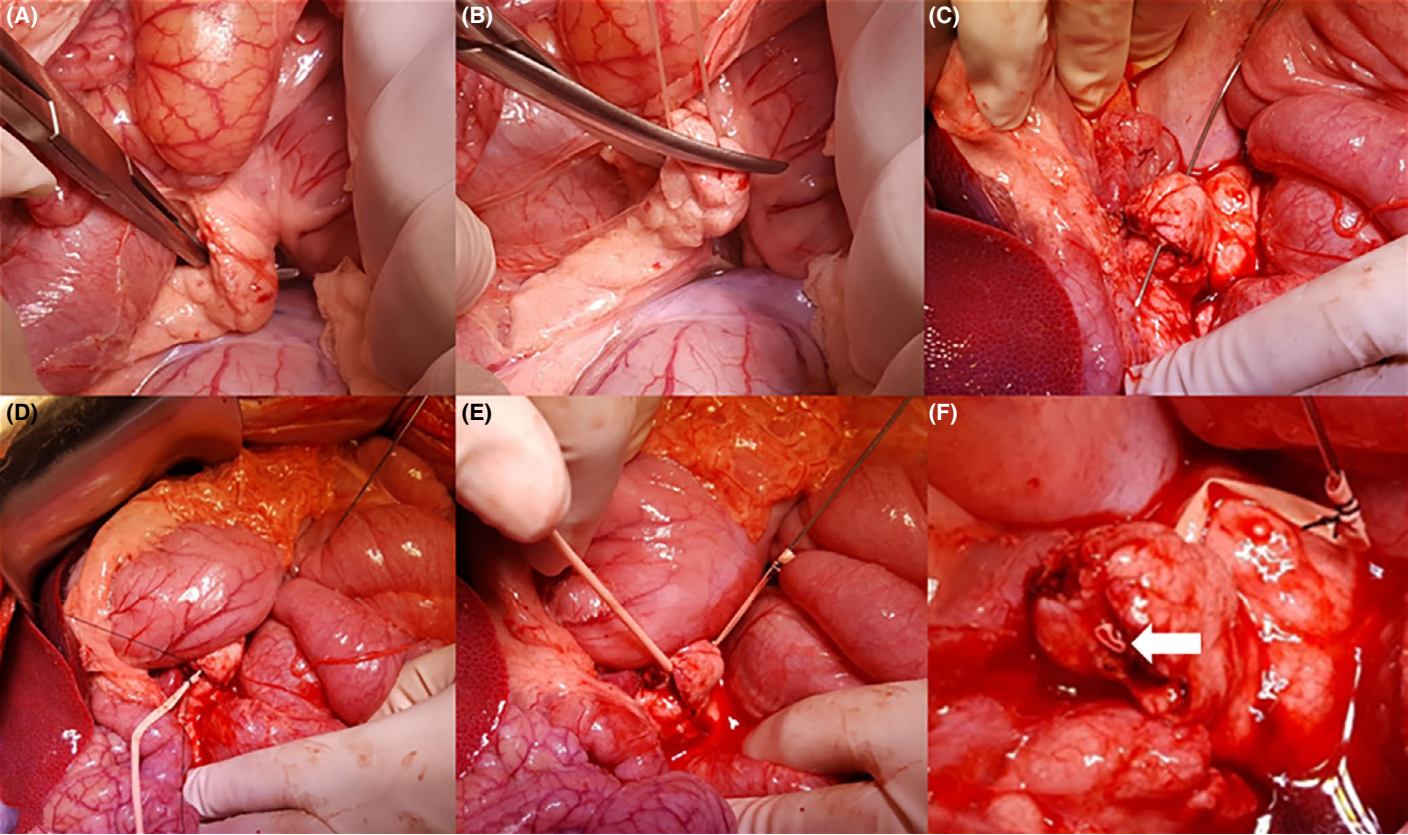
Steps for constructing a *Yonsei‐PJ^DTM^ model*. A, Dissect the pancreatic neck of the SMV‐SV‐PV confluence. B, Divide the pancreatic neck. C, Insert a curved wire or catheter from the dorsal part of the left pancreatic neck portion. D, Fix a small rubber tube (artificial neo‐pancreatic duct) to the wire. E, Withdraw the wire to implant a small rubber tube as a neo‐pancreatic duct into the soft pancreas. F, Final view of the *Yonsei‐PJ^DTM^ model.* The lumen size of the neo‐pancreatic duct can be determined according to trainee's requirement. A 1‐2‐mm catheter will be appropriate for simulating a challenging clinical situation. An implanted artificial neo‐pancreatic duct can be seen in the cut surface of the pancreas (white arrow)

To facilitate neo‐artificial pancreatic duct implantation into the left‐side pancreas, a specially designed catheter can be applied. The end of this catheter divides into two branches; 1.5 cm from the endpoint of the catheter is a 5 mm hole at an angle of 45° so that it can exit above the pancreas. Further details are filed with the Korean Intellectual Property Office (Figure 1).^8^ The catheter can be inserted simply using the following steps (Figure 2).

## 3 RESULTS

### 3.1 Surgical simulation using the Yonsei‐PJ^DTM^ model

**Figure 3:**
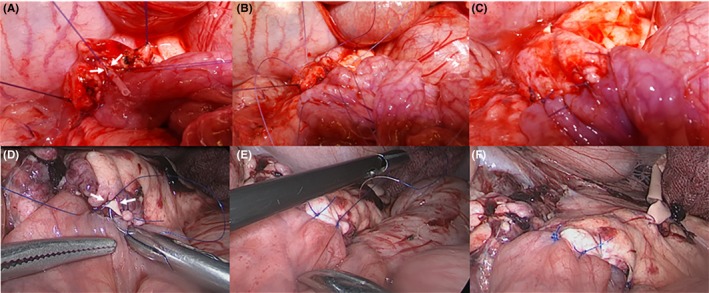
Simulating open and laparoscopic PJ using the Yonsei‐PJ^DTM^ model. Posterior interrupted sutures and duct‐to‐mucosa anastomosis (A and D). Note an implanted artificial pancreatic duct (white arrow). Anterior interrupted sutures (B and E), and a final view of PJ (C and F)

There are various techniques for PJ during PD.^9^, ^10^ In our clinical practice, an interrupted, DTM (4‐6 stitches), two‐layer technique with a short stent is useful for managing soft pancreas with a shorter than 2 mm pancreatic duct. This procedure can be simulated with either an open or laparoscopic approach (Figure 3). After completing PJ, dividing the distal part of the pancreas near the PJ can provide a new model for repeated PJ training. The preparation of a porcine model for PJ (Yonsei‐PJ^DTM^) and surgical simulation are well‐described in the Video S1.

## 4 DISCUSSION

Managing soft remnant pancreas with a small pancreatic duct during PD is challenging.^5^ This step is an Achilles heel for safe PD. Therefore, appropriate preoperative surgical rehearsal is necessary for enhancing the safety of PD. Considering the increase in minimally invasive PD, this unmet clinical need should be resolved for safe implementation of minimally invasive PD in clinical practice.^11^

We developed an in vivo porcine model mimicking remnant soft pancreas with a small pancreatic duct during PD. A specialized catheter may be very helpful for preparing this model,^12^ although a typical catheter or curved guide wire can be used.

To our knowledge, there is no appropriate surgical simulation model for DTM PJ in remnant soft pancreas with a small pancreatic duct. Currently, only in vitro and ex vivo simulation models have been developed.^12^, ^13^

The following are potential advantages of the proposed porcine model of PJ (Yonsei‐PJ^DTM^). First, the present model can provide similar conditions to soft remnant pancreas with a small pancreatic duct. Therefore, using this model, most surgical techniques for PJ can be simulated. Second, each porcine model can be used for at least three or four PJ simulations. Third, the size of the neo‐pancreatic duct can be changed according to lumen size using an artificial rubber tube. Fourth, this model can be applied not only in open surgery, but also in laparoscopic or robotic surgery. After preparing an in vivo porcine model using the open approach, minimally invasive surgical simulation can be performed after closing the abdominal wound. Lastly, a combination of bile duct isolation, transection, and choledochojejunostomy (this module is not presented in this report but has been introduced and shown to be reproducible^14^) will be a good surgical training module for simulating the reconstruction phase of PD.

Minimally invasive PD is reported to be safe and feasible.^1^, ^3^, ^15^ However, safe implementation of a surgical technique for minimally invasive PD should be based on appropriate and reproducible surgical simulation for practicing surgical skill. Establishing a proper surgical educational program for safe PD requires further investigation. Before performing PJ in patients, it would be beneficial if less experienced surgeons could learn the concepts of the surgical process (lectures), observe video clips (video library) or expert operations showing skillful surgical techniques, and perform in vitro surgical simulation. As a final stage of preoperative surgical rehearsal, the present in vivo porcine model can play a potential role in improving minimally invasive (laparoscopic or robotic) PJ for safe PD.

This study has several limitations. First, it is the first to construct a porcine model; there is no accumulated data to support the efficiency of this proposal for training with laparoscopic PJ. We are currently collecting data and will report it in the future. Second, this proposal has the advantage of practicing multiple PJ simulations with one experimental pig, although it is expensive to prepare the pig. Much funding will be needed for training in minimally invasive surgery, and systematic help of the local hepato‐biliary‐pancreas association is critical.

In conclusion, the Yonsei‐PJ^DTM^ model could be an option for training on PJ, and further study is needed.

## DISCLOSURE

Funding: This study was supported by the InSuk (Chi Hoon Sang) Best Teacher Award (2015) of Severance Surgeon's Alumni, Department of Surgery, Yonsei University College of Medicine, Seoul, Korea.

Conflict of Interest: Chang Moo Kang has Korea Patent 1018637860000 about this technique, filed March 02, 2017, by Industry‐Academic Cooperation Foundation, Yonsei University, issued May 28, 2018.

Author Contribution: Munseok Choi acquired and drafted the manuscript. Chang Moo Kang conceived and designed the study, revised the manuscript, and gave final approval to the manuscript.

## References

[1] Peng L , Zhou Z , Cao Z , Wu W , Xiao W , Cao J . Long‐term oncological outcomes in laparoscopic versus open pancreaticoduodenectomy for pancreatic cancer: a systematic review and meta‐analysis. J Laparoendosc Adv Surg Tech A. 2019;29(6):759–69. 30835156 10.1089/lap.2018.0683

[2] Navarro JG , Kang CM . Pitfalls for laparoscopic pancreaticoduodenectomy: need for a stepwise approach. Ann Gastroenterol Surg. 2019;3(3):254–68. 31131354 10.1002/ags3.12242 PMC6524087

[3] Kang CM , Lee SH , Chung MJ , Hwang HK , Lee WJ . Laparoscopic pancreatic reconstruction technique following laparoscopic pancreaticoduodenectomy. J Hepatobiliary Pancreat Sci. 2015;22(3):202–10. 25546026 10.1002/jhbp.193

[4] Eshmuminov D , Schneider MA , Tschuor C , Raptis DA , Kambakamba P , Muller X , et al. Systematic review and meta‐analysis of postoperative pancreatic fistula rates using the updated 2016 International Study Group Pancreatic Fistula definition in patients undergoing pancreatic resection with soft and hard pancreatic texture. HPB (Oxford). 2018;20(11):992–1003. 29807807 10.1016/j.hpb.2018.04.003

[5] Nagakawa Y , Nakamura Y , Honda G , Gotoh Y , Ohtsuka T , Ban D , et al. Learning curve and surgical factors influencing the surgical outcomes during the initial experience with laparoscopic pancreaticoduodenectomy. J Hepatobiliary Pancreat Sci. 2018;25(11):498–507. 30291768 10.1002/jhbp.586

[6] Bailey KL , Carlson MA . Porcine models of pancreatic cancer. Front Oncol. 2019;9:144. 30915276 10.3389/fonc.2019.00144 PMC6423062

[7] Choi SH , Choi JJ , Kang CM , Hwang HK , Lee WJ . A dog model of pancreaticojejunostomy without duct‐to‐mucosa anastomosis. JOP. 2012;13(1):30–5. 22233944

[8] Industry‐Academic Cooperation Foundation, Yonsei University and Chang moo Kang, An apparatus for producing an animal model for pancreaticojejunostomy practice, an animal model produced by the apparatus, and a method for producing the animal model. Korea Patent 10‐2017‐0027181, filed March 02, 2017, and issued June 01, 2018.

[9] Kakita A , Yoshida M , Takahashi T . History of pancreaticojejunostomy in pancreaticoduodenectomy: development of a more reliable anastomosis technique. J Hepatobiliary Pancreat Surg. 2001;8(3):230–7. 11455485 10.1007/s005340170022

[10] Lee WJ . Fish‐mouth closure of the pancreatic stump and parachuting of the pancreatic end with double U trans‐pancreatic sutures for pancreatico‐jejunostomy. Yonsei Med J. 2018;59(7):872–8. 30091321 10.3349/ymj.2018.59.7.872 PMC6082987

[11] van Hilst J , de Rooij T , Bosscha K , Brinkman DJ , van Dieren S , Dijkgraaf MG , et al. Laparoscopic versus open pancreatoduodenectomy for pancreatic or periampullary tumours (LEOPARD‐2): a multicentre, patient‐blinded, randomised controlled phase 2/3 trial. Lancet Gastroenterol Hepatol. 2019;4(3):199–207. 30685489 10.1016/S2468-1253(19)30004-4

[12] Avila R , Achurra P , Tejos R , Buckel E , Rebolledo R , Sanhueza M , et al. Simulated training for the acquisition of skills in the laparoscopic pancreaticojejunostomy. HPB. 2017;19:S41.

[13] Narumi S , Toyoki Y , Ishido K , Kudo D , Umehara M , Kimura N , et al. Introduction of a simulation model for choledocho‐ and pancreaticojejunostomy. Hepatogastroenterology. 2012;59(119):2333–4. 22687967 10.5754/hge11544

[14] Lee JS , Hong TH . In vivo porcine training model for laparoscopic Roux‐en‐Y choledochojejunostomy. Ann Surg Treat Res. 2015;88(6):306–10. 26029675 10.4174/astr.2015.88.6.306 PMC4443261

[15] Croome KP , Farnell MB , Que FG , Reid‐Lombardo KMarie , Truty MJ , Nagorney DM , et al. Total laparoscopic pancreaticoduodenectomy for pancreatic ductal adenocarcinoma: oncologic advantages over open approaches? Ann Surg. 2014;260(4):633–8; discussion 8‐40. 25203880 10.1097/SLA.0000000000000937

